# Epigenetic regulation of long non-coding RNAs in gastric cancer

**DOI:** 10.18632/oncotarget.23821

**Published:** 2017-12-16

**Authors:** Zhixia Zhou, Zhijuan Lin, Xin Pang, Muhammad Akram Tariq, Xiang Ao, Peifeng Li, Jianxun Wang

**Affiliations:** ^1^ Center for Tumor Molecular Biology, Institute for Translational Medicine, Qingdao University, Qingdao 266021, China

**Keywords:** lncRNAs, gastric cancer, epigenetic regulation, DNA methylation, histone modification

## Abstract

Gastric cancer is one of the most common cancers and is the second leading cause of cancer mortality worldwide. Therefore, it is urgent to explore new molecular biomarkers for early diagnosis, early treatment and prognosis for gastric cancer patients. Recently, increasing evidence has shown that epigenetic changes, such as aberrant DNA methylation, histone modifications, and noncoding RNAs (ncRNAs) expression, play substantial roles in the development and progression of malignancies. Among these changes, long non-coding RNAs (lncRNAs), a novel class of ncRNAs, are emerging as highly versatile actors in a variety of cellular processes by regulating gene expression at the epigenetic level as well as at the transcriptional and post-transcriptional levels. Hundreds of lncRNAs become dysregulated in the various pathological processes of gastric cancer, and multiple lncRNAs have been reported to function as tumor-suppressors or oncogenes, although the underlying mechanisms are still under investigation. Here, we provide an overview of the epigenetic regulation of chromatin and the molecular functions of lncRNAs; we focus on lncRNA-mediated epigenetic regulation of cancer-related gene expression in gastric cancer, as well as discuss the clinical implications of lncRNAs on epigenetic-related cancer treatments, which may contribute helpful approaches for the development of new potential strategies for future diagnosis and therapeutic intervention in human cancers.

## INTRODUCTION

Gastric cancer (GC) is one of the most frequently diagnosed gastrointestinal neoplasms, which has significantly affected a large population worldwide, especially in East Asia [1, 2]. Although the mortality rate of GC has declined significantly with recent advances in chemotherapy, radiotherapy and surgical techniques, the overall survival rate remains less than 25% once gastric cancer metastasizes [3, 4]. This low survival rate is mainly because GC typically grows slowly over the years, and GC is generally undetectable at early stages due to the lack of novel molecular biomarkers for diagnosis [5, 6]. Similar to other cancers, the molecular mechanisms of GC are very complex and poorly understood. Genetic alterations, epigenetic changes and environmental factors are major contributors to the development and progression of malignancies [7, 8]. Genetic mutations contribute to the loss of function or differential expression of proteins involved in metabolic pathways, whereas epigenetic changes play key roles in the dysregulation of oncogenes and tumor suppressor genes; both gene types are involved in cancer initiation and progression [3, 7, 8]. Multiple epigenetic mechanisms, including DNA methylation, hydroxymethylation, posttranslational modifications of histone proteins, chromatin remodeling and noncoding RNAs (i.e., long noncoding RNAs (lncRNAs)), govern the reduced expression or overexpression of genes, such as DNA repair genes, cell cycle regulators, apoptotic genes, transcriptional regulators, and signaling pathway regulators, during the development and progression of GC [7, 8]. The relentless changes in these molecular machineries subsequently cause uncontrolled cell proliferation, migration, invasion, and resistance to apoptosis in GC cells. Environmental factors, including *Helicobacter pylori* infection [9], Epstein-Barr Virus infection [10], high salt consumption [6], hypoxic stress [11], smoking and erratic lifestyle [12], also contribute to the development of the disease. The environmental impact on GC incidence can be assessed and reduced; however, epigenetic changes may only be controlled and/or managed via a thorough understanding of epigenetic regulation [10].

### Epigenetic regulations

Epigenetics is defined as the study of heritable changes in DNA that affect the packaging of chromatin, and these changes are not caused by any modification in the primary DNA sequence [13, 14]. These changes could regulate the ability of the cell’s own transcription machinery to express certain genes from a particular section of chromatin, and these changes may also be lost for multiple generations [13]. However, no changes are noted in the underlying DNA sequence of the organism, which is different from mutations that can result in different types of sequence changes [15]. Genomic imprinting is an inherited epigenetic phenomenon by which certain genes are expressed in a parent-of-origin-specific manner depending on which allele inherited from a parent [16]. The imprinted genes are marked by discrete elements termed imprinting control regions (IRCs), which play a critical role in the imprinting of multiple genes [17]. Genomic imprinting occurs in the germline (sperm or egg cells) of the parents, and the imprinted status is maintained through mitotic cell divisions in the somatic cells of an organism [18]. Integrated retroviral DNA represents an important agent of disease and serves as a valuable vector for gene delivery. However, this DNA is also subject to epigenetic transcriptional silencing at different frequencies. Thus, the epigenetic regulation of chromatin plays an important role in the regulation of gene expression in normal growth, embryonic development, X-Chromosome inactivation, and even disease initiation.

### DNA methylation

DNA methylation is the first-discovered and best-characterized epigenetic modification and typically occurs in CpG islands (cytosine nucleotide bases followed by guanine bases) and CG rich regions located upstream of the promoter region. DNA methylation is a major epigenetic factor involved in the regulation of tissue-specific gene expression, genomic imprinting, X-chromosome inactivation and silencing of retroviral elements [19]. Particularly, DNA methylation in different genomic regions differentially influences gene activities. Based on genetic sequencing studies, methylation of intergenic regions, the promoter region, or the *5′* region of the gene has been associated with gene silencing at the transcriptional level [19, 20]. DNA methylation can also alter the condensed chromatin structure by influencing histone-DNA or histone-histone contact. DNA methylation is catalyzed by a group of enzymes called DNA methyltransferases (DNMTs) that transfer methyl groups from S-adenosylmethionine to the C5 position of cytosine. Several different DNA methyltransferases have been described, such as DNMT1, DNMT3A, DNMT3B, and DNMT3L. DNMT1 maintains methylation during DNA replication, whereas DNMT3A and 3B are important for the establishment of *de novo* DNA methylation [21]. DNMT3L is a regulatory protein that is catalytically inactive for methyl transfer but is involved gene repression independent of DNA methylation [22].

### Histone modification

Histone modification is another extensively studied epigenetic modification that involves several different covalent modifications of histone N-terminal tails, such as acetylation, methylation, phosphorylation, ubiquitination, and sumoylation [23, 24]. Among them, acetylation and methylation are the most general and important histone modifications associated with transcriptional activation of gene expression [25]. Histone acetylation is catalyzed by histone acetyl transferases (HATs), whereas the reverse reaction is mediated by histone deacetylases (HDACs) [25]. HATs promote gene transcription by neutralizing positive charges of chromosomal elements to open the chromatin and promote subsequent transactivation of specific genes. HDACs promote chromatin condensation and transcriptional inactivation [26, 27]. Generally, histone H3 is primarily acetylated at several lysine residues, including Lys9, 14, 18, 23, 27 and 56, whereas histone H4 is acetylated at Lys5, 8, 12 and 16 [25]. Histone methylation is a complex process by which methyl groups are transferred to the amino acids of histone proteins in chromosomes. Histones differentially influence transcription of genes depending on which amino acid is methylated and the degree of methyl groups (mono, di or tri) attached. Histone methylation also often occurs at lysine and arginine residues in histones H3 and H4 via processes regulated by histone methyltransferases (HMTs) and histone demethylases (HDMs) [28]. In fact, the chromatin structure can be opened by trimethylation at H3K4 and H3K36 or closed by trimethylation at H3K27, H3K9, and H4K20 and dimethylation at H3K9. These effects further depend on the type of residue and the level of methylation [29].

### Chromatin remodeling

Chromatin remodeling is a dynamic modification of chromatin architecture to overcome the barrier presented by chromatin structures to the regulatory transcription machinery proteins [30]. This function relies on a group of enzymes termed chromatin remodeling complexes, which are classified into five families (SWI/SNF, ISWI, CHD, INO80 and SWR1 families) depending on the type of ATPase subunit present in the complexes [31]. Each chromatin remodeling family catalyzes ATP-dependent restructuring and repositioning of nucleosomes. These proteins operate as histone octamer-anchored directional DNA translocases to disrupt DNA-histone interactions or catalyze nucleosome sliding. Chromatin remodeling plays a vital role in regulating gene expression and activation, DNA replication and repair, apoptosis, chromosome segregation, developmental processes and pluripotency [32].

### Non-coding RNAs (ncRNAs)

ncRNAs are functional RNA molecules that are transcribed from DNA but not translated into proteins. ncRNAs are key regulators of chromatin structure in eukaryotic cells in addition to their role in RNA degradation and translational repression. ncRNAs are implicated in controlling gene expression and chromatin modification *via* RNA interference (RNAi) pathways. According to their location, length, structure, or biological functions, ncRNAs can be classified into different categories. For instance, canonical ncRNAs, such as ribosomal RNAs (rRNAs) and transfer RNAs (tRNAs), were discovered earlier based on their functions in protein synthesis. In contrast, small RNAs (sRNAs), small nucleolar RNAs (snoRNAs) and small nuclear RNAs (snRNAs) are found in specific cellular locations [33]. Other ncRNAs are mainly classified based on the length of their mature products. These RNAs can be short (< 200 nt) or long (> 200 nt) and are further classified based on genomic origin and mechanism of action. ncRNAs involved in the regulation of gene expression through epigenetic mechanisms are divided into two main groups: long ncRNAs (lncRNAs) and short ncRNAs, which include microRNAs (miRNAs), short interfering RNAs (siRNAs), and PIWI-interacting RNAs (piRNAs) [34–36].

miRNAs regulate gene expression through cleavage, degradation, or blockage of translation of specific target mRNAs by imperfect pairing with the *3’*-UTR region of target genes, which may also involve DNA methylation mechanisms [37, 38]. Similarly, siRNAs bind to target mRNAs with complementary nucleotide sequences and cause post-transcriptional gene silencing (PTGS) by guiding the mRNAs to the degradation process. siRNAs also mediate transcriptional gene silencing by inducing heterochromatin formation *via* histone methylation and chromatin condensation [39]. piRNAs are named based on their interaction with the PIWI family of proteins, and their primary function is to maintain germline integrity and fertility. piRNAs are produced *via* a Dicer-independent mechanism and mediate gene silencing by binding to PIWI proteins. As antisense to transposon sequences, piRNAs are primarily involved in transposon silencing by cleaving the transposon through PIWI-protein complexes [40]. The PIWI-piRNA complex also contributes to histone modifications by activating methylation processes. These findings suggest that piRNAs guide PIWI and PIWI-associated epigenetic factors for epigenetic programming of the genome [41]. LncRNAs represent the major (70–90% of the human genome) ncRNA transcripts. Many lncRNAs can complex with chromatin-modifying proteins and recruit their catalytic activity to specific sites in the genome, thereby modifying chromatin states and influencing gene expression [42, 43].

As mentioned above, these epigenetic mechanisms occur in various pathological processes during the development of gastric cancer. In this review, we summarized the up-to-date research progress on lncRNA and lncRNA-associated epigenetic regulation in gastric cancer. The advancement in our understanding about the underlying mechanisms of epigenetic alterations and the influence of lncRNAs in these mechanisms could lead to the identification of potential clinical biomarkers for the diagnosis, prognosis, treatment, and drug development for GC cancers.

## LNCRNAS AND THEIR FUNCTIONS

LncRNAs have long been considered transcripts without protein-coding capacity. Recent studies have demonstrated that lncRNAs have a limited ability to encode information for proteins [3] potentially because lncRNAs share many characteristic features with mRNAs as follows: *1)* Both mRNA and lncRNA are transcribed by RNA polymerases (Pol I, II, or III) and undergo splicing, polyadenylation, and *5*-capping [44]; *2)* LncRNAs also have the ‘K4-K36 domain’ of active promoters (H3K4me2/3, H3K9ac, H3K27ac) and actively transcribed gene bodies (H3K36me3), which are well-characterized epigenetic markers of chromatin for mRNAs [45]; *3)* Some lncRNAs possess similar sequences, such as the *3’* UTR region of mRNA, with respect *to* their secondary structures, sequence composition and thermodynamic parameters [46, 47]. However, various distinctive features of lncRNAs distinguish this class of RNAs from mRNA. A majority of lncRNAs are localized in the nucleus. LncRNAs lack or have small open reading frames (ORFs) and exhibit poor sequence conservation. Although most lncRNAs are normally expressed at lower levels than mRNAs, they exhibit more specific expression patterns in cells and tissues [42, 48].

It is being increasingly recognized that lncRNAs are involved in a variety of biological functions during evolutionary conservation, selection and development, including chromosomal dosage compensation, chromatin imprinting, chromatin modification, maintenance of chromatin structure, transcription, splicing and translation [49, 50]. The execution of these functions is based on at least four archetypes: *1)* Signals, as molecular signals depending on their specific cell or tissue expression patterns; *2)* Decoys, as a ‘molecular sink’ binding a protein to titrate it away without exerting any function; *3)* Guides, to direct the localization of the ribonucleoprotein resulting in the expression of genes loading in *cis* or *trans* of lncRNAs; *4)* Scaffolds, to control the intermolecular interactions and signaling events by providing platforms for different effector molecules [49, 50]. Based on the growing evidence of lncRNA functionality, more archetypes will be uncovered, and it is not a surprise that lncRNAs have been implicated in different human diseases.

LncRNAs are differentially expressed in various human diseases, including cancer [36], heart failure [51], diabetes mellitus [51] and neuropsychiatric disorders [52]. Thus, lncRNAs are emerging as key players in disease development and progression. LncRNA dysfunction leads to alterations in gene regulatory networks that consequently lead to disease transformation, such as aberrant changes in proliferation, differentiation, and apoptosis [7]. Based on their various genomic locations (intergenic regions, intronic regions, antisense regions, promoter regions, and UTR regions) and their possible origins (from the ORFs of a protein-coding gene, two separated sequences juxtapose following the rearrangement of a chromosome, non-coding gene retrotransposition, tandem duplication events or insertion of a transposable element) [42, 49, 53], lncRNAs broadly regulate gene expression through diverse mechanisms. Three prominent levels of gene regulation by lncRNAs are noted. First, lncRNAs regulate target genes at the transcriptional level by impeding the association of transcription factors, enhancers or promoters through interacting with DNA to form triple helix structures or RNA PolII [49, 54]. LncRNAs can also act as co-activators or endogenous competitive RNAs. Second, lncRNAs regulate target genes at the post-transcriptional levels (including splicing, transport, translation, and degradation) by cutting mRNAs into small ncRNAs, interacting with mRNAs to form double-stranded RNAs, altering the alternative splicing of pre-mRNAs, or interacting with other types of ncRNAs [3, 49]. Third, lncRNAs regulate target genes at the epigenetic level by shaping the epigenome through regulating DNA methylation, histone modification, genomic imprinting, and chromatin remodeling or by interacting with other ncRNAs, especially miRNAs [49, 55]. In recent years, our understanding of the mechanisms of gene regulation by lncRNAs has been increasing. In particular, the epigenetic gene regulation by lncRNAs is widely studied by numerous researchers. A deep understanding about the epigenetic regulation of disease-related lncRNAs is critical to recognize their function in the prevention, diagnosis, prognosis and treatment of diseases, including cancer.

## LNCRNA-ASSOCIATED EPIGENETIC REGULATION IN GASTRIC CANCER

The dysregulation of oncogenes and tumor suppressor genes due to multiple genetic and epigenetic alterations is considered a driving force of tumorigenesis, including gastric cancer. LncRNA-mediated epigenetic regulation of cancer-related gene expression is often associated with epigenetic mechanisms, such as DNA methylation, DNA hydroxymethylation, post-translational modifications of histone proteins, or regulation of miRNAs [5]. Interestingly, the epigenetic mechanisms not only alter the expression of cancer-related genes via associations with lncRNA, but the expression of lncRNAs is also regulated by epigenetic mechanisms, including DNA methylation, histone modifications and miRNAs, as presented in Table 1.

**Table 1:** The epigenetic regulation lncRNAs in gastric cancer

LncRNA	Expression	Mechanism	Target	Reference
AK058003	Upregulation	Hypoxia; Methylation in the CpG islands	SNCG	[59]
AK123072	Upregulation	Hypoxia; Methylation in the CpG islands	EGFR	[60]
HOTTIP	Upregulation	Methylation in the CpG islands; H3K4 methylation	HoxA13	[61, 62]
HOXA11-AS	Upregulation	DNA methylation; Histone modification; miR-1297	E2F1; P21; E-cadherin; EZH2	[63, 64]
HOTAIR	Upregulation	H3K27 trimethylation; miR-34a	C-Met; Snail	[68–70]
MALAT1	Upregulation	H3 histone acetylation	EGFL7	[36, 71]
GClnc1	Upregulation	Histone modification	SOD2	[72]
LINC00673	Upregulation	SP1; Histone modification	KLF2; LATS2	[73]
AGAP2-AS1	Upregulation	SP1; Histone modification	P21; E-cadherin	[74]
ZFAS1	Upregulation	Histone modification	NDK2; KLF2	[75]
PVT1	Upregulation	Histone modification	p15; p16	[70]
LINC00668	Upregulation	E2F1; Histone methylation	p15; p16; p21; p27; p57	[76, 77]
TUG1	Upregulation	Histone methylation	P57	[78]
LINC00152	Upregulation	Histone methylation	p15; p21	[79]
FEZF1-AS1	Upregulation	H3K4 methylation	p21	[80]
ANRIL	Upregulation	Histone modification; miR-99a/miR-449a	p15INK4B; p16INK4A; mTOR; CDK6	[83–85]
SPRY4-IT1	Downregulation	DNA methylation	SPRY4-IT1	[87]
LOC100130476	Downregulation	Methylation in the CpG islands	LOC100130476	[88]
GAS5	Downregulation	Promoter methylation	GAS5	[89, 90]
FENDRR	Downregulation	Histone deacetylation	FENDRR	[90, 91, 92]
MEG3	Downregulation	Promoter methylation; miR-148a	MEG3; RUNX3	[93, 94, 95–97]

### LncRNAs regulate DNA methylation

The regulation of DNA methylation by lncRNAs is an important mechanism that controls gene expression during cancer progression and patient outcome. Differential expression of lncRNAs has been reported in cancer initiation, progression, and metastasis. For example, lncRNA-ecCEBPA, a novel lncRNA encoded by the *CEBPA* gene locus, is critical for the prevention of *CEBPA* gene methylation and robust *CEBPA* mRNA production in a leukemic cell through the association with DNMT1 [56]. HOTAIR is a lncRNA transcribed from the HOX locus that affects the chromatin methylation state of the HOXD locus through the recruitment of the polycomb repressive complex (PRC2). In laryngeal squamous cell carcinoma cells, HOTAIR is overexpressed and promotes CpG methylation in the promoter region of the tumor suppressor gene *PTEN*, resulting in the loss of *PTEN* in cancer cells [57]. Linc-POU3F3 is a lncRNA that is approximately 4-kb upstream from the transcription factor *POU3F3* gene, which promotes cell viability and proliferation in esophageal squamous cell carcinoma cells. These oncogenic effects of the linc-POU3F3 are related its cooperation with EZH2 to regulate the methylation level of its neighboring gene, and alterations in linc-POU3F3 levels accelerate *POU3F3* gene methylation [58].

**Figure 1:**
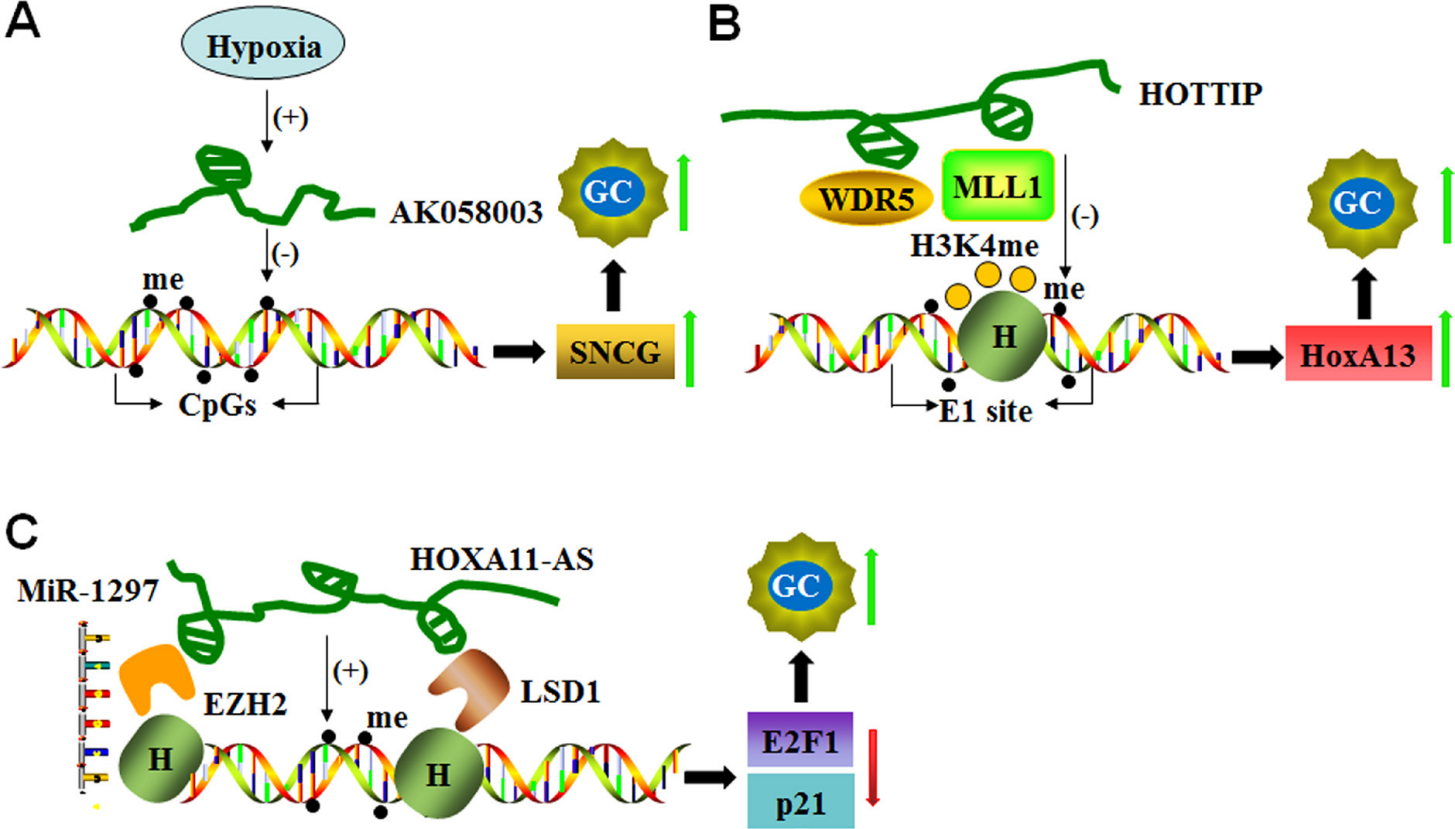
LncRNAs mediate DNA methylation of target genes (**A**) Hypoxia-induced AK058003 up-regulates *SNCG* expression by decreasing DNA methylation in its CpG islands and promoting GC metastasis and invasion. (**B**) HOTTIP increases *HoxA13* expression to enhance GC progression not only by down-regulating DNA methylation at the E1 site but also by recruiting MLL1 and WDR5 to control histone H3K4 methylation at the E1 site. (**C**) HOXA11-AS functions as a scaffold and recruits EZH2, LSD1 and DNMT1 to decrease *E2F1* and *p21*. HOXA11-AS also functions as a molecular sponge for miR-1297 and antagonizes its repressive function on EZH2 translation, which results in cell cycle progression and cell proliferation in GC.

In GC cells and tissues, the expression of lncRNA-AK058003, a 1197-bp transcript located on the forward strand of the chromosome 10q22, is strongly induced by hypoxia. This lncRNA enhances GC cell migration and invasion by regulating the expression of *SNCG*, a metastasis-associated gene [59]. *SNCG* mRNA levels are positively correlated with the expression of lncRNA-AK058003 in clinical GC samples. Under hypoxic conditions, lncRNA-AK058003 up-regulates *SNCG* expression by decreasing CpG island methylation in *SNCG*. A gene knockdown study confirmed that lncRNA-AK058003 silencing down-regulates *SNCG* expression at the mRNA level [59] (Figure 1A). These findings indicate that hypoxia/lncRNA-AK058003/*SNCG* is a new signaling pathway involved in GC metastasis and invasion.

AK123072 is an intronic antisense lncRNA that is often up-regulated in GC. AK123072 promotes GC migration and invasion *via* increasing *c-Myc* mRNA stability and expression. Similar to lncRNA-AK058003, AK123072 also increases GC cell metastasis by targeting EGFR under hypoxic conditions. AK123072 knockdown significantly increases the methylation of CpG islands in the *EGFR* gene, thereby down-regulating its expression [60].

LncRNA HOTTIP (HOXA transcript at the distal tip) is located at the *5’* tip of the HOXA locus and coordinates the activation process of several *5’ HOXA* genes by recruiting PRC2 and WD repeat domain 5 (WDR5)–mixed lineage leukemia (MLL) complex to the *5*′-end of the HOXA cluster, resulting in H3K4 methylation and transcriptional activation of the HOXA locus [61]. HOTTIP is highly expressed in the human GC cell line CS12, and HOTTIP knockdown reduces the expression of *5’*-end *HOXA* genes, including *HoxA13*. Increased expression of the *HoxA13* gene and its downstream cascades is involved in the tumorigenic activity of CS12 cells. A molecular study demonstrated that the recruitment of MLL1 and WDR5 and the methylation of histone H3 occurs at the CpG position (designated as the E1 site; a composite *p53*/*E2F*-binding site) of the *HoxA13* promoter in CS12 cells. In addition, reduced DNA methylation at this site is also observed with the restriction of DNMT1 and DNMT3b recruitment to the E1 site [62]. HOTTIP suppression restores the recruitment of DNMT3b but not DNMT1. HOTTIP silencing also reduces WDR5 and MLL1 recruitment and the level of trimethylation of histone H3 lysine 4 (H3K4me3) at the site [62] (Figure 1B).

HOXA11 antisense RNA (HOXA11-AS) is a lncRNA that is an important determinant of cancer progression. Gastric cancer patients with high HOXA11-AS expression exhibit reduced survival and poorer prognosis. Silencing of HOXA11-AS suppresses cell growth, migration, and invasion and promotes apoptosis by interfering with *E2F1*, *CDKN1A* (*P21*), and *E-cadherin* transcription [63, 64]. In addition, HOXA11-AS acts as a scaffold by recruiting enhancer of zest homolog 2 (EZH2) along with DNMT1 [64], suggesting that DNMT1-mediated DNA methylation is involved in HOXA11-AS function (Figure 1C).

### LncRNAs regulate histone modification

**Figure 2:**
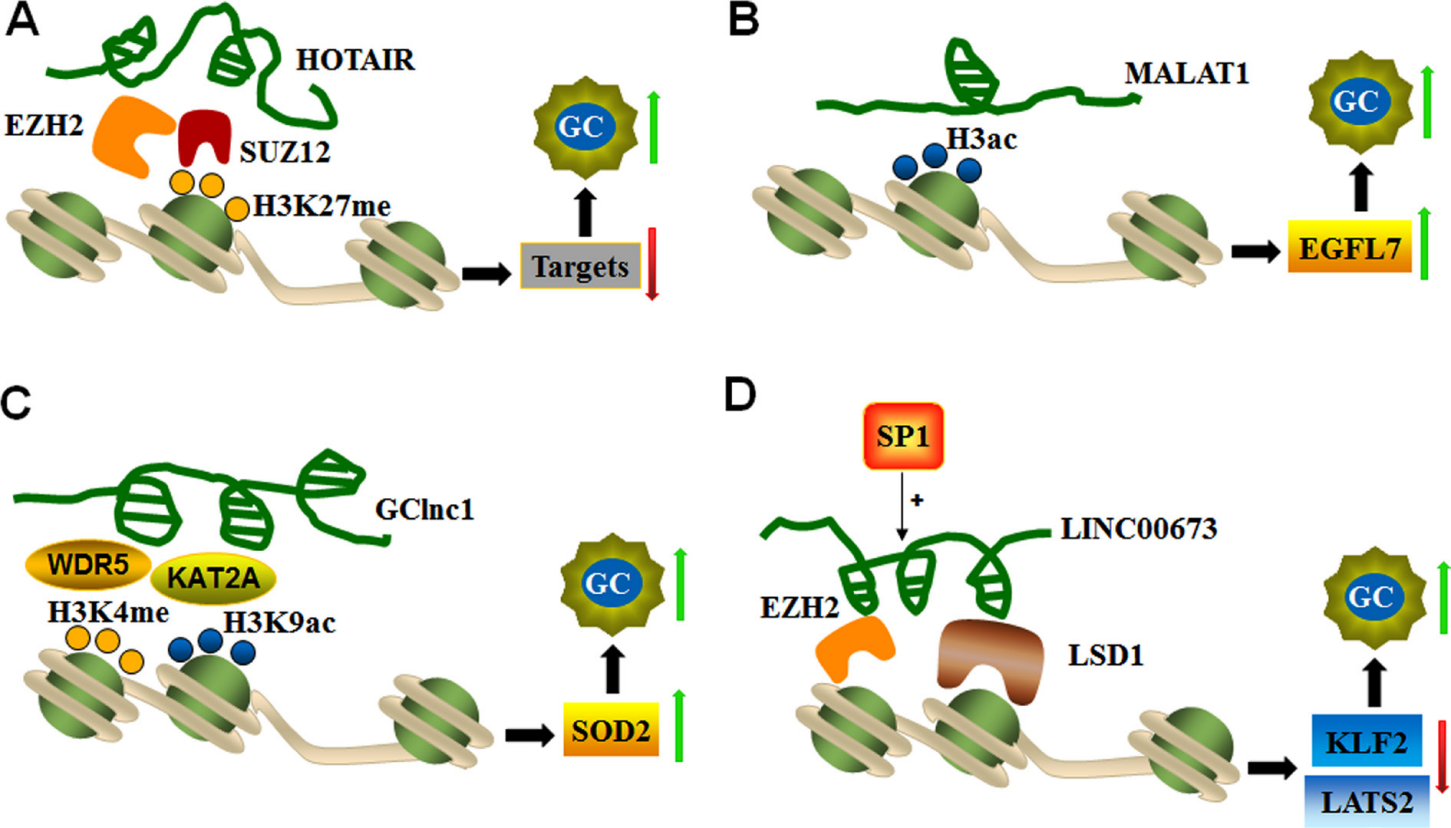
LncRNAs mediate histone modifications of target genes (**A**) HOTAIR recruits EZH2 and the SUZ12 complex to silence target genes *via* H3K27 trimethylation and thereby promotes GC progression. (**B**) MALAT1 up-regulates *EGFL7* expression, which is related to GC invasion and migration by altering the level of H3 histone acetylation in its promoter region. (**C**) GClnc1 coordinates WDR5 and KAT2A localization and specifies the histone modification pattern to increase SOD2 expression during GC tumorigenesis. (**D**) *SP1*-activated LINC00673 represses KLF2 and LATS expression by functioning as a scaffold for LSD1 and EZH2 to enhance GC cell proliferation and invasion.

LncRNAs guide chromatin-modifying complexes to specific genomic loci and influence gene expression. For instance, HOTAIR is the first lncRNA identified to interact with PRC2 and the LSD1/CoREST/REST complex through its *5’*- and *3’*-ends, respectively [65]. PRC2 is the main chromatin regulatory factor and is composed of methylase EZH2, SUZ12, and EED. PRC2 is involved in trimethylation of specific lysine (K) residues of K9 and K27 in histone H3 (H3K27me3) [66], whereas the LSD1 complex serves as a demethylase that mediates histone H3 lysine 4 demethylation (H3K4me2). HOTAIR mediates the assembly of the PRC2 and LSD1 complex and enables the targeting of specific gene loci to induce H3K27me3 and H3K4me2 modification [65, 67]. Overexpression of HOTAIR is noted in various cancerous tissues, including colorectal cancer, pancreatic cancer, hepatocellular carcinoma, and nasopharyngeal carcinomas. HOTAIR is responsible for regulating epigenetic cellular memory and cancer development [65, 67, 68]. In gastric tumors, high HOTAIR expression is potentially associated with high TNM staging and lymph node metastasis [68, 69]. In addition, a strong positive correlation is noted between HOTAIR and SUZ12 expression levels in gastric adenocarcinoma tissues. SUZ12 is part of PRC2 and binds with RNA molecules through the zinc-finger domain during chromatin silencing. These findings suggest that HOTAIR promotes genomic relocalization of PRC2 and H3K27 trimethylation in gastric cancer [68]. Furthermore, EZH2 (a subunit of PRC2) and SUZ12 knockdown reduces EZH2 and H3K27 binding ability, which confirms the interaction between HOTAIR and PRC2. These findings indicate that HOTAIR recruits the PRC2 complex to silence target gene *via* H3K27me3 modification in GC progression [70] (Figure 2A).

The novel MALAT1 (metastasis-associated lung adenocarcinoma transcript 1) transcript represents a highly conserved lncRNA in mammals that is expressed from chromosome 11q13. MALAT1 consists of greater than 8000 nt and is associated with multiple types of physiological processes, such as alternative splicing, nuclear organization, and epigenetic modulation of gene expression [71]. MALAT1 is highly expressed in GC tissues and cell lines. MALAT1 expression is related to cell invasion and migration by promoting the expression of epidermal growth factor-like domain-containing protein 7 (EGFL7). MALAT1 increases *EGFL7* expression by altering the level of H3 histone acetylation in the *EGFL7* promoter region [36], as shown in Figure 2B.

GClnc1 is a newly identified lncRNA in gastric cancer. GClnc1 is up-regulated and associated with tumorigenesis, tumor size and metastasis. GClnc1 is involved in poor prognosis by acting as a modulating scaffold of WDR5 (a key component of histone methyltransferase complex) and KAT2A histone acetyltransferase (Figure 2C). GClnc1 coordinates WDR5 and KAT2A localization and specifies the histone modification pattern of the target gene *SOD2*, indicating that GClnc1 is functionally an oncogenic factor in gastric cancer [72].

HOTTIP is another highly expressed lncRNA in human gastric CS12 cancer cells. HOTTIP regulates *HoxA13* gene expression by recruiting DNA methyltransferase (DNMT3b) and affecting *HoxA13* histone modification as described above (Figure 1B). This study indicates that HOTTIP is indeed involved in the epigenetic regulation of the *HoxA13* gene in gastric cancer cells.

As a gastric cancer-associated lncRNA, HOXA11-AS is a key regulator of gastric cancer development and progression. HOXA11-AS silencing causes alterations in cell proliferation and cell–cell adhesion pathways. Mechanistically, EZH2 and LSD1 (histone demethylase) are recruited by HOXA11-AS, which acts as a scaffold to form the EZH2/HOXA11-AS/LSD1 axis, a critical effector in gastric cancer tumorigenesis and progression (Figure 1C) [64].

LncRNA-LINC00673 is an intergenic lncRNA located on chromosome 17q25.1 that is significantly up-regulated in gastric cancer. LINC00673 knockdown inhibits cell proliferation and invasion and induces cell apoptosis, thus revealing the role of LINC00673 in oncogenesis. *SP1* activates LINC00673 transcription by directly binding to its promoter region. A molecular study found that LINC00673 interacts with EZH2 and LSD1, and this complex is involved in the repression of *KLF2* and *LATS2* expression (Figure 2D). These findings indicate that *SP1*-activated expression of LINC00673 promotes GC development and progression by functioning as a scaffold for LSD1 and EZH2 and repressing the expression of tumor suppressor genes, such as *KLF2* and *LATS2* [73].

AGAP2-AS1 is an antisense lncRNA transcribed from a gene located at 12q14.1. AGAP2-AS1 is highly expressed in GC tissues with poorer prognosis and reduced overall survival. AGAP2-AS1 knockdown significantly inhibits GC cell proliferation, migration, and invasion. Similar to LINC00673, there are several *SP1* binding sites in the promoter regions of AGAP2-AS1, and *SP1* can bind to all of these sites in the promoter region of AGAP2-AS1 to induce AGAP2-AS1 expression. Furthermore, AGAP2-AS1 epigenetically suppresses *P21* and *E-cadherin* expression by interacting with EZH2 and LSD1, indicating that AGAP2-AS1 also has oncogenic roles in gastric cancer [74].

LncRNA-ZFAS1 (zinc finger antisense 1) is a newly identified lncRNA, and its expression is up-regulated in gastric cancer tissues and cells. LncRNA-ZFAS1 overexpression is associated with poor prognosis and reduced survival. ZFAS1 contributes to tumorigenesis by increasing cell proliferation and resisting cancer cell apoptosis. The oncogenic effects of ZFAS1 are partially mediated by epigenetic silencing of *KLF2* and *NKD2* expression through recruiting EZH2 and LSD1 to the *NDK2* and *KLF2* promoter regions. This action consequently represses their transcription by increasing trimethylation of histone H3 at lysine 27 (H3K27me3) and demethylation of H3K4me2 [75]. These findings demonstrate that *ZFAS1* acts as an oncogene and a negative prognostic factor in gastric cancer patients.

**Figure 3:**
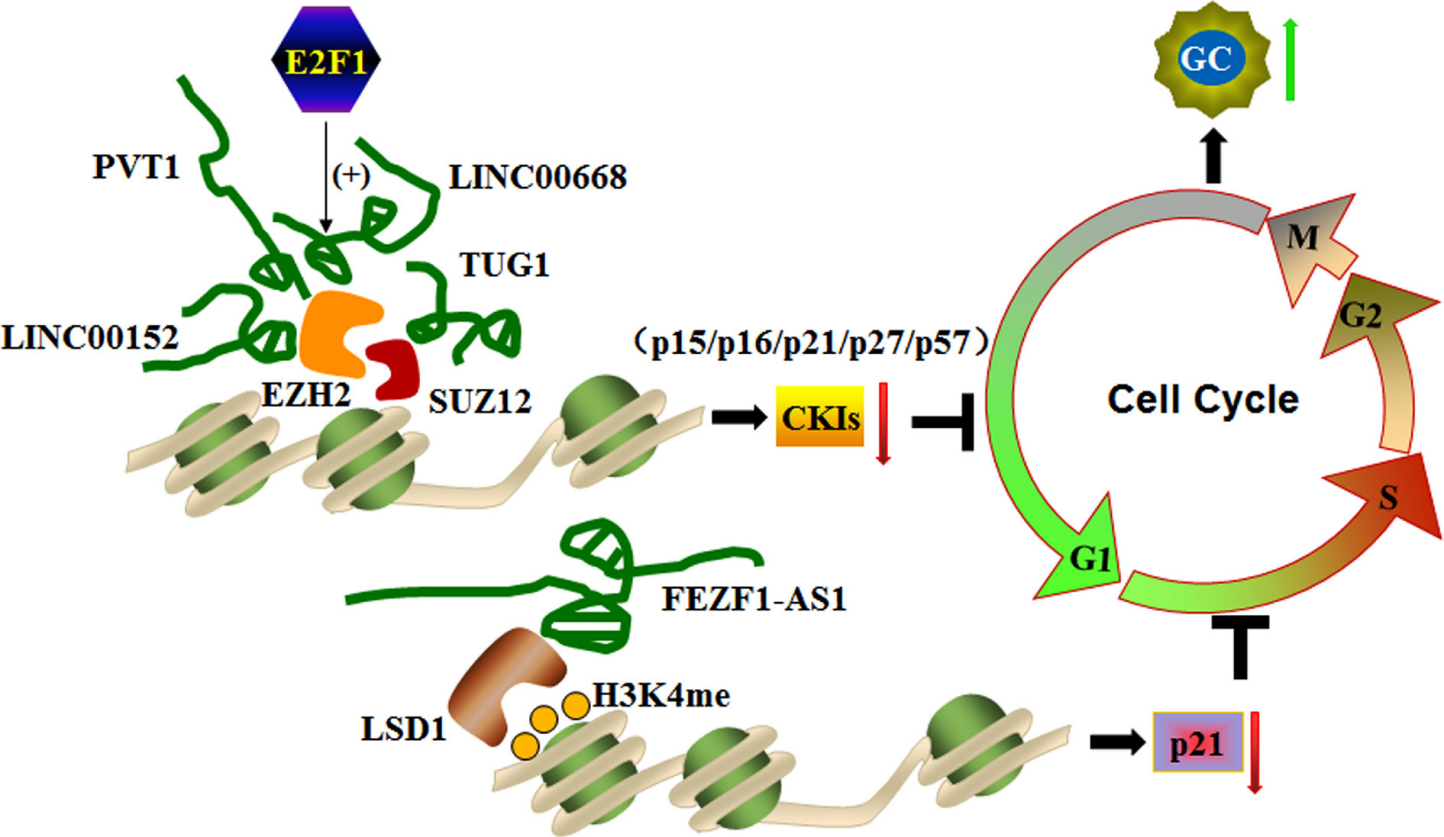
LncRNAs silence cyclin-dependent protein kinase inhibitors (CKIs) by interacting with histone modification machineries to control the cell cycle *Sp1*-activated LINC00668, PVT1, LINC00152, and TUG1 promote the G0/G1 phase of the cell cycle to enhance gastric cancer cell proliferation and tumorigenesis by binding to EZH2 or SUZ12 and silencing *CKIs*, including *P12*, *p16*, *p21*, *p27*, and *p57*. In addition, EZF1-AS1 promotes the G1/S phase of the cell by repressing *p21* transcription through recruiting LSD1 and causing H3K4me2 demethylation at the gene promoter region.

LncRNAs regulate cell cycle in gastric cancer cells by epigenetically silencing several cyclin-dependent protein kinase inhibitors (*CKIs*), including *p15*, *p16*, *p21*, *p27* and *p57*. LncRNAs, such as PVT1 (Plasmacytoma variant translocation 1), LINC00668, LINC00152, TUG1 and FEZF1-AS1, contribute to cell cycle progression in GC cells (Figure 3).

PVT1 is identified at a reakpoint site in chromosome 6 translocations. PVT1 expression is up-regulated in human GC tissues and cell lines and correlates with poor prognosis in GC. PVT1 associates with the EZH2 enhancer to suppress *p15* and *p16* expression, which induces cell cycle arrest and controls cell cycle progression. A gene knockdown study confirmed this finding. PVT1 silencing reduces cell proliferation by inducing G1 arrest and promoting malignant cell apoptosis, which results in attenuation of tumorigenesis [70]. These findings indicate that PVT1 activates PRC2-mediated epigenetic suppression of *p15* and *p16* and promotes GC cell proliferation.

LINC00668 is a 1751-bp long noncoding RNA identified in gastric cancer. *E2F1* acts as a transcription factor for the expression of LINC00668, which plays a pivotal role in cell cycle progression. High LINC00668 expression is noted in GC, and its expression is positively correlated with invasion depth, TNM stage, and poor prognosis. LINC00668 enhances gastric cancer cell proliferation by binding to PRC2 and activating EZH2 to silence *CKIs*, partially accounting for LINC00668-mediated cell growth regulation [76]. PRC2-mediated histone methylation contributes to the repression of *CKIs* [77]. This study reveals that *E2F1*-induced up-regulation of LINC00668 down-regulates *CKI* expression via histone methylation and thereby promotes malignant cell proliferation [76].

Similar to LINC00668, TUG1 (the first identified lncRNA in a screen for genes up-regulated by developing retinal cells in response to taurine) and LINC00152 (an intergenic lncRNA located in the chromosome 2) are also unregulated and well correlated with outcomes in numerous cancers, including gastric cancer. Inhibition of TUG1 or LINC0015 expression represses GC proliferation by arresting the cell cycle at the G0/G1 phase. Both TUG1 and LINC0015 bind to PRC2, and this association is required for silencing of *CKIs,* including *p15*, *p16*, *p21*, *p27* and *p57* [78, 79]. Taken together, these findings suggest that PRC2-mediated histone methylation and epigenetic silencing of *CKIs* are governed by the lncRNAs LINC00668, TUG1 or LINC00152. Thus, this process controls the expression of *CKIs* and promotes cell cycle progression in gastric cancer cells.

FEZF1-AS1 is another newly identified lncRNA in gastric cancer. FEZF1-AS1 is a 2564-bp transcript located on chromosome 7. FEZF1-AS1 is overexpressed in gastric cancer tissues, and its expression is correlated with poor prognosis. FEZF1-AS1 represses *p21* transcription by recruiting LSD1 and facilitating H3K4me2 demethylation in the promoter region of *p21*, which is one of the most important *CKIs* that activates the checkpoints of *P53* signaling pathway involved in inhibition of cyclin-dependent kinase activity. In this context, FEZF1-AS1 promotes malignant cell proliferation in advanced stages of gastric cancer. FEZF1-AS1 knockdown significantly represses proliferation and inhibits cells cycle progression by arresting gastric cancer cells at the G1/S phase, confirming its role in gastric cancer development and progression. These findings suggest that FEZF1-AS1 has oncogenic potential in GC cells [80].

### LncRNAs regulate miRNA expression and activity

Emerging evidence reveals that lncRNAs can also serve as a competing endogenous RNAs (ceRNAs) to antagonize the effects of miRNAs on their target mRNAs. Therefore, the lncRNA-miRNA-mRNA network plays a pivotal role in tumorigenesis and metastasis similar to other oncogenes or tumor suppressor genes [81, 82].

**Figure 4:**
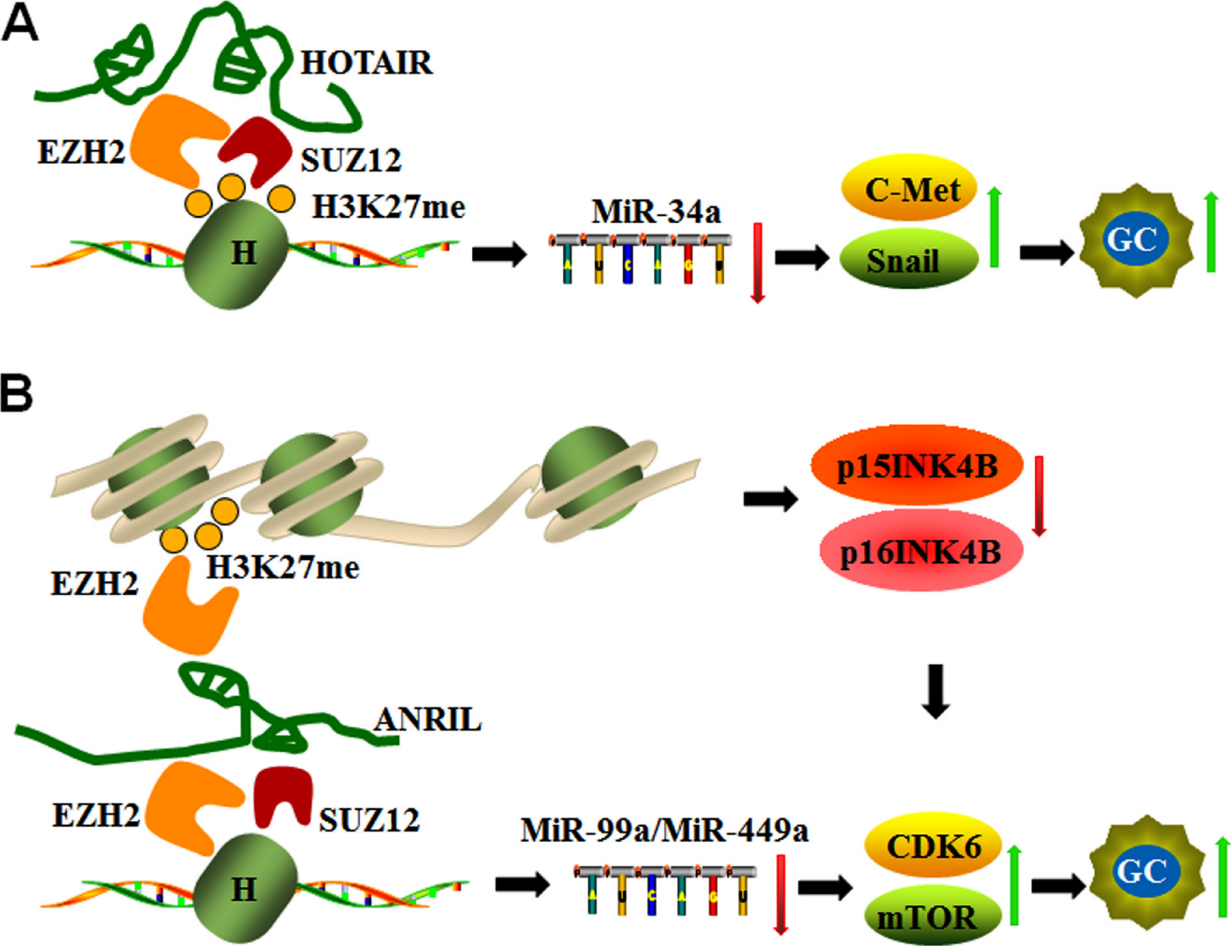
LncRNAs act as inhibitors or sponges for epigenetic regulation of miRNAs (**A**) HOTAIR silences miR-34a by binding to EZH2 and SUZ12, mediating H3K27 methylation and subsequently activating C-Met and Snail during GC progression. (**B**) ANRIL not only down-regulates miR-99a/miR-449a expression by binding EZH2 and SUZ12 in *trans* but also regulates *mTOR* and *CDK6/E2F1* pathways by silencing *p15INK4B* and *p16INK4A* expression (*CDK6* inhibitors) *via* EZH2 binding and H3K27 trimethylation in *cis*, which promotes *CDK6* activation and GC cell proliferation.

miR-34a expression is down-regulated and negatively correlated with *HOTAIR* expression in gastric cancer tissues. The co-ordination of HOTAIR and PRC2 down-regulates miR-34a expression and activates miR34a target genes, such *C-Met* (*HGF/C-Met/Snail* pathway) and Snail, which are central players in the epithelial-mesenchymal transition (EMT) in advanced stages of gastric cancer. miR-34a expression is up-regulated in knockdown models of EZH2 (also a subunit of PRC2) and SUZ12. Molecular studies confirmed that EZH2 directly binds to the promoter region of miR-34a and induces H3K27me3 modification, whereas knockdown of HOTAIR and EZH2 reduced EZH2 binding to H3K27 (Figure 4A). These studies indicate that HOTAIR recruits the PRC2 complex to silence miR34a expression *via* H3K27me3 modification during GC progression [70].

ANRIL, which is also known as CDKN2B-AS1, is an antisense ncRNA of the INK4 locus that is transcribed from the *INK4A*-*ARF*-*INK4B* gene cluster in the opposite direction and functions as an oncogene in cancers. ANRIL promotes cell cycle progression and resistance to apoptosis via epigenetic silencing of tumor suppressor genes *p15INK4B* and *p16INK4A* through binding with EZH2 and trimethylation of H3 (H3K27) in *cis* [83, 84]. In addition, ANRIL epigenetically silences miR-99a/miR-449a expression in GC cells by binding to PRC2 with EZH2 and SUZ12 in *trans*, and this complex triggers *mTOR* and *CDK6/E2F1* pathways to promote GC cell proliferation and growth [85]. Given that *CDK6* is a target of miR-449a that can also be inhibited by *p15INK4B* and *p16INK4A*, the positive feedback loop formed by ANRIL promotes GC cell proliferation [85] as shown in Figure 4B.

In gastric cancer, HOXA11-AS not only acts as a scaffold for EZH2, LSD1 and DNMT1 but also functions as a molecular sponge for miR-1297 to antagonize its repressive effect on EZH2 translation [64]. In addition, the transcription factor *E2F1* activates HOXA11-AS expression, which up-regulates the expression of genes involved in the cell cycle and cell cycle progression from G1 into S phase [64] (Figure 1C). These findings suggest that HOXA11-AS/miR-1297/EZH2 cross-talk plays a critical role in cell growth, migration, invasion, and apoptosis.

### Epigenetic regulation of lncRNA expression

**Figure 5:**
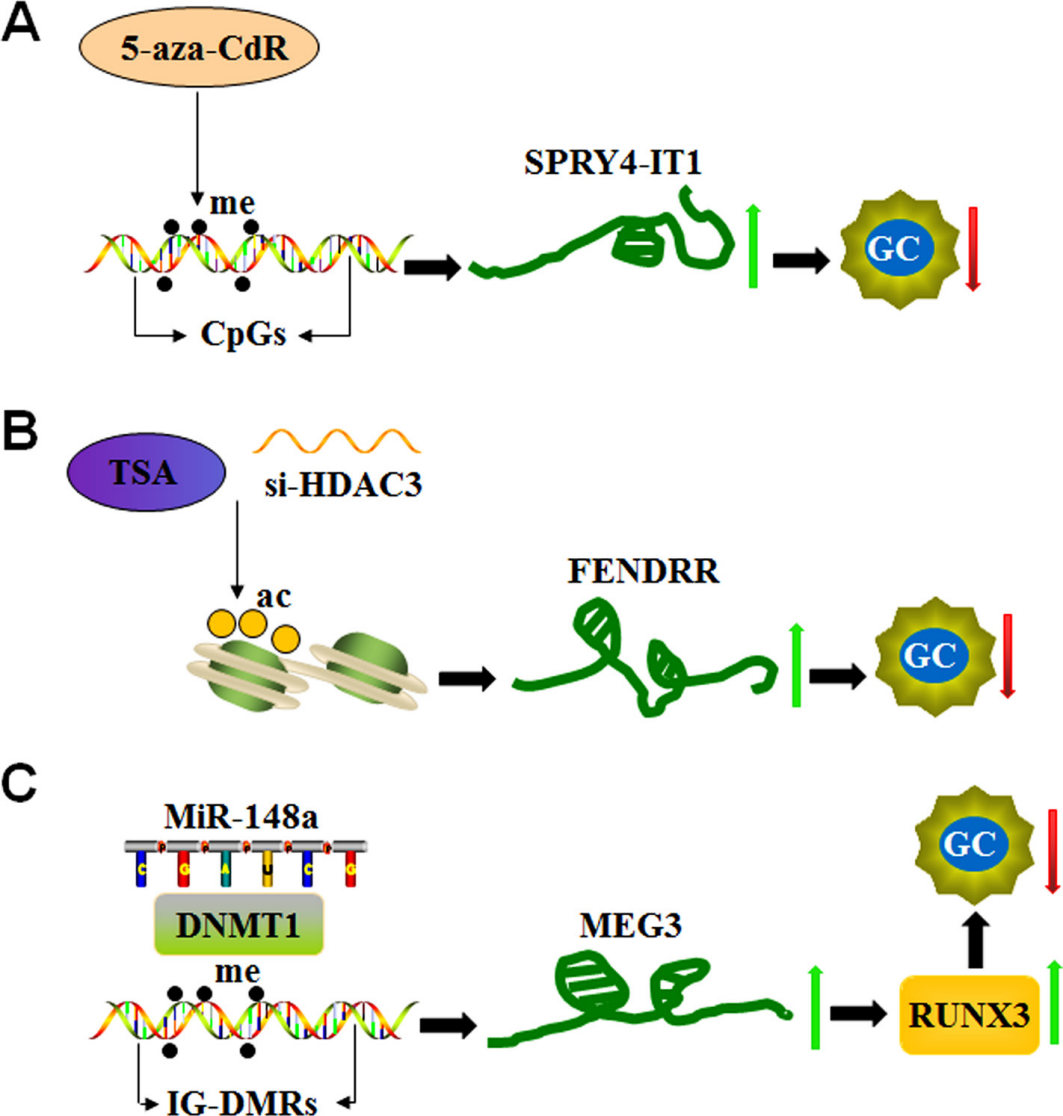
LncRNA expression is epigenetically regulated in GC (**A**) The DNA methyltransferase inhibitor 5-aza-CdR increases SPRY4-IT1 expression by targeting CpG islands in its promoter region and inhibiting the development of GC. (**B**) The histone deacetylase (HDAC) inhibitor trichostatin A (TSA) or HDAC3 small interfering RNAs (si-HDAC3) induces FENDRR expression by targeting histone deacetylation and reducing cell invasion and migration in GC. (**C**) MiR-148a up-regulates MEG3 expression by recruiting DNA methyltransferase 1 (DNMT1) and decreasing methylation of the MEG3 regulatory regions (IG-DMRs), consequently reducing tumor size in GC patients.

The expression of numerous lncRNAs is up-regulated in response to treatment with DNA methylation inhibitors in gastric cancer cells. This finding indicates that lncRNA expression can be regulated by the methylation process [86]. For instance, the expression of lncRNAs, such as SPRY4-IT1, LOC100130476, GAS5 (growth arrest-specific transcript 5) and MEG3 (maternally expressed gene 3), is modified by DNA methylation in GC (Figure 5).

SPRY4-IT1 is a lncRNA derived from an intron of the *SPRY4* gene, which is down-regulated in gastric cancer tissues. The level of reduction of SPRY4-IT1 is associated with tumor size, advanced pathological stage, depth of invasion, lymphatic metastasis and poor prognosis given its role in the regulation of EMT process [87]. A genomic study identified canonical CpG islands in the promoter region of the SPRY4-IT1 loci and that a DNA methylation inhibitor (5-aza-CdR) significantly increases its expression in GC cells. In addition, knockdown of DNMT1 up-regulates SPRY4-IT1 expression [87]. These findings highlight that DNMT1-mediated DNA methylation represents a vital factor in controlling SPRY4-IT1 expression (Figure 5A).

LOC100130476 is a lncRNA located on 6q23.3 that is down-regulated in GC tissues and cells. LOC100130476 expression is associated with TNM stage, pathological differentiation, and survival in GC patients [88]. The methylation frequency of LOC100130476 gradually increases from exon 1 to exon 2. The methylation status of region 1, which is close to the transcription start site, is more tumor-specific, indicating that the aberrant methylation of CpG sites near the transcription start site within exon 1 is critical for LOC100130476 expression and consequently causes poor prognosis [88].

The expression level of growth arrest-specific transcript 5 (GAS5) lncRNA is significantly down-regulated in gastric cancer tissues, and its reduction is associated with cancer cell migration and invasion [89, 90]. In addition, GAS5 expression is further decreased in Adriamycin (ADM)-resistant gastric cancer cells, and increased methylation levels are noted in the GAS5 promoter region. The DNA methylation inhibitor 5-AZA-dC significantly reduces the methylation status of the GAS5 gene. GAS5 expression is also restored by epigenetic inhibitors, which subsequently results in reduction in the cancer cell growth rate and increased apoptosis. These reports suggest that GAS5 is down-regulated in gastric cancer cells by promoter hypermethylation, which further regulates Adriamycin sensitivity [89].

Histone modifications also contribute to the regulation of lncRNA expression. For instance, FENDRR lncRNA (FOXF1 adjacent noncoding developmental regulatory RNA) binds to PRC2 and Trithorax group/Mixed lineage leukemia complexes to control chromatin structure and gene activity. These complexes play an important role in the differentiation of lateral mesoderm and heart and body development [90, 91]. Reduced FENDRR expression is observed in GC, and its reduction is associated with tumor invasion, advanced tumor stage, lymphatic metastasis, poor prognosis and invasion depth. However, FENDRR expression does not correlate with tumor size. FENDRR overexpression effectively reduces the cell invasion and migration mediated by fibronectin1 and MMP2/MMP9. Notably, two CpG islands are located in the promoter region and the first exon of FENDRR. However, FENDRR expression is not significantly altered in GC cells upon treatment with the DNA methyltransferase inhibitor (5-aza-C), which indicates that DNA methylation does not significantly contribute to control FENDRR expression. Similarly, the knockdown of two core subunits of PRC2 (SUZ12 and EZH2) does not influence FENDRR expression. Interestingly, FENDRR expression is induced by the histone deacetylase (HDAC) inhibitor trichostatin A (TSA) and HDAC3 small interfering RNAs (si-HDAC3) (Figure 5B), indicating that histone deacetylation machineries, in particular HDAC3 enzymatic activity, is involved in the down-regulation of FENDRR in GC cells [92].

MEG3 is located on human chromosome 14q32.3 and encodes a tumor suppressor lncRNA. The promoter region and the intergenic germ line-derived differentially methylated region (IG-DMR) of MEG3 are rich in CpG dinucleotides [93]. This finding indicates that promoter methylation plays an important role in the loss of MEG3 expression in tumors (Figure 5C). In this context, MEG3 levels are markedly reduced in gastric cancer tissues, and its reduction significantly correlates with TNM stage, depth of invasion and tumor size. Similar to SPRY4-IT1, MEG3 expression is up-regulated by 5-aza-CdR treatment, indicating that reduced MEG3 levels in gastric cancer cells could be partly due to hypermethylation of the IG-DMR of the MEG3 gene [94]. In addition, a positive correlation is noted between MEG3 and miR-148a expression levels (Figure 5C). miR-148a suppresses tumorigenesis through regulating DNA methyltransferase 1 expression [95]. miR-148a modulates the expression of runt-related transcription factor 3 (RUNX3), an important tumor suppressor, through inhibition of DNMT1-dependent DNA methylation in gastric cancer [96]. These reports suggest that miR-148a suppression contributes to MEG3 down-regulation in gastric cancer by DNMT-1 activation [97].

## CONCLUDING REMARKS

In recent years, epigenetic changes have been demonstrated to be the main factors involved in tumorigenesis and cancer development. Remarkable progress has been made to utilize epigenetic machineries to develop treatments for cancer by reversing the epigenetic abnormalities occurring in tumors. For example, various drugs targeting epigenetic pathways, including DNMT inhibitors and HDACs inhibitors, are experimentally and clinically effective in cancer treatment. However, the underlying mechanisms of epigenetic changes in tumors have not been completely investigated, and further studies are required to identify more specific and effective agonists or inhibitors for epigenetic treatment. Given the tissue and disease specificity of the expression pattern of lncRNAs and their ability to control the epigenetic regulatory network, lncRNAs have gained considerable importance not only as biomarkers but also as therapeutic targets for treating tumors. Several laboratories are currently investigating the potential use of lncRNAs for diagnostic, prognostic, and curative powers for various cancers, including GC. Currently, numerous available techniques support the feasibility of using lncRNA-based therapeutics. In the case of down-regulated metastasis-suppressing lncRNAs, their level can be improved using liposomes/nanoparticles to deliver plasmid-based expression vectors or viral-based expression vectors. To inhibit overexpressing metastasis-promoting lncRNAs, RNAi/shRNAs or CRISPR-Cas9-dependent interference can be used. Nevertheless, considerable efforts are needed to obtain the most effective and least harmful epigenetic treatments for cancer. In-depth studies of epigenetic changes, especially lncRNAs, are expected to enhance the efficiency of lncRNA usage as potential clinical biomarkers and therapeutic targets in the treatment of cancer.

## Abbreviations

ADM: adriamycin
ANRIL: antisense non-coding RNA in the INK4 locus
ceRNA: competing endogenous RNA
CKI: cyclin-dependent protein kinase inhibitor
DNMT: DNA methyltransferases
EGFL7: epidermal growth factor-like domain-containing protein 7
EMT: epithelial-mesenchymal transition
EZH2: recruiting enhancer of zest homolog 2
FENDRR: FOXF1 adjacent noncoding developmental regulatory RNA
GAS5: growth arrest-specific transcript 5
GC: gastric cancer
HAT: histone acetyltransferase
HDAC: histone deacetylase
HDM: histone demethylase
IG-DMR: intergenic germline-derived differentially methylated region
IRC: imprinting control region
LncRNAs: long noncoding RNA
miRNA: microRNAs
MALAT1: metastasis associated lung adenocarcinoma transcript 1
MEG3: maternally expressed gene 3
MLL: mixed lineage leukemia
H3K4me: histone H3 lysine 4 demethylation
HMT: histone methyltransferase
ncRNA: non-coding RNA
ORF: open reading frame
piRNA: PIWI-interacting RNA
PRC2: polycomb repressive complex
PVT1: plasmacytoma variant translocation 1
RNAi: RNA interference
rRNAs: ribosomal RNAs
RUNX3: runt-related transcription factor 3
snoRNAs: small nucleolar RNAs
snRNAs: small nuclear RNAs
siRNA: short interfering RNA
sRNAs: small RNAs
tRNAs: transfer RNAs
TSA: trichostatin A
TUG1: taurine up-regulated gene 1
WDR5: WD repeat domain 5
